# The Child Attachment Interview: A Narrative Review

**DOI:** 10.3389/fpsyg.2017.00384

**Published:** 2017-03-14

**Authors:** Antonella Privizzini

**Affiliations:** Dipartimento Salute Mentale e Dipendenze, ASL 3 GenoveseGenova, Italy

**Keywords:** Child Attachment Interview, middle childhood, adolescence, mentalization, child development

## Abstract

Attachment theory promoted an impressive body of research on the psychic developmental processes, resulting in studies on both typical and atypical development. Much of the diffusion of the attachment theory in the clinical field was related to the design of reliable instruments to evaluate the organization of attachment in infancy as well as in adulthood. Until recently, the lack of a suitable instrument to assess attachment in middle childhood as well as in adolescence hindered the expansion of research in these developmental phases during which the parent-child relationship takes on a different, albeit still crucial, role. The Child Attachment Interview (CAI), a measure that was recently designed to assess attachment at a representational level in middle childhood and adolescence, filled the measurement gap. The aim of the current review was to summarize previous empirical investigations concerning CAI in order to (a) provide an overview of the state of current research, (b) identify unanswered questions, and (c) propose future research directions. A narrative review was conducted to map the current research findings by searching for the term “Child Attachment Interview” in the Medline, Scopus, Web of Science, and PsychINFO databases, followed by a search in Mendeley. Limits were set to exclude dissertations, chapters in books, and qualitative or theoretical papers, while empirical studies were included if they used the CAI and were published in English language, peer-reviewed journals by July, 2016. The review, which ultimately included 39 studies meeting the criteria, showed that the CAI is a reliable instrument to assess attachment organization in clinical and non-clinical samples, thus providing a worthwhile contribution to the investigation of the influence of the parent-child relationship beyond infancy and early childhood. Nevertheless, the review pointed out a number of relevant open issues, the most critical of which concerned the CAI coding and classification system. In particular, some relevant questions arose about (a) how opportune it would be to maintain a distinct classification for mother and father, (b) coding challenges regarding both the father and the Preoccupied and Disorganized classification, and finally (c) the advantage of a dimensional vs. a categorical approach.

## Introduction

Attachment theory promoted an impressive body of research on the psychic developmental processes, resulting in studies on both typical and atypical development. Much of the diffusion of the attachment theory in the clinical field, as well as its remarkably quick spread in the scientific community through a myriad of studies, was possible because of the design of reliable instruments to evaluate the organization of attachment in infancy as well as in adulthood (e.g., Ainsworth et al., 1978; George et al., 1985). This made it possible to empirically investigate the influence of the parent-child relationship on the psychic development of the child, especially concerning self-organization, and the interpersonal sphere, particularly social cognition (Fonagy et al., 2007).

Until recently, the lack of a suitable instrument to assess attachment in middle childhood as well as in adolescence hindered the expansion of research in these developmental phases during which the parent-child relationship takes on a different, albeit still crucial, role (e.g., Allen, 2008).

The Child Attachment Interview (CAI; Shmueli-Goetz et al., 2000), a measure designed to assess attachment at a representational level, especially in middle childhood, and which was recently extended to adolescence (Venta et al., 2014), filled the measurement gap. Measuring attachment in middle childhood posed some challenges, in that attachment could no longer be assessed through behavioral measures as is the case in infancy and in early childhood. At the same time, it was questionable whether children in middle childhood are able to fully represent their attachment organization solely through verbal communication. Thus, the CAI was developed by integrating both the behavioral and the representational approach. Behavioral analysis allows us to observe behavioral changes in response to a particular question, as well as anxiety, maintaining eye contact, tone of voice, discrepancy between behavior and contents of the narrative, and general behavioral adequacy. The CAI was developed on the basis of the Adult Attachment Interview (AAI; George et al., 1985), in that it requires the interviewee to describe his/her relationship with each parent and to support the general description with relevant episodic memories, but it is different to some extent in that the child is asked to talk about his/her current relationships. It was designed to grasp the child's state of mind regarding attachment independently with respect to the mother and the father. Furthermore, it activates the attachment system since it requires the child to talk to a stranger about his/her closest private relationships. The most recent version of the CAI protocol (Target et al., 2007) includes 19 questions plus probes regarding self-description, a description of the relationship with each parent, the episodes in which parents became cross with the child as well as those during which they argued with each other, and the times in which the child felt upset or experienced illnesses, separations, and loss.

The CAI transcript is evaluated by assigning a score ranging from 1 (the lowest score) to 9 (the highest score) to the following nine scales: Emotional Openness, Balance of positive/negative references to attachment figures, Use of examples, Preoccupied Anger, Resolution of conflicts, Idealization, Dismissal, Atypical/Disorganized behavior, and Overall coherence.

Emotional Openness considers the breadth of the range of feelings the child is able to contemplate and the degree to which he/she is able to include them in the relational episodes that he/she tells, taking into account the interaction of affects and behavior. The lowest rate is assigned when the transcript contains no mention or illustration of affect, whereas the highest score is assigned when the CAI narrative is affectively laden with detailed illustrations.

The “Balance of positive/negative references to attachment figures” subscale evaluates to what extent the child is able to take into account both positive and negative aspects of her/his parents as well as of his/her relationship with them.

“Use of examples” assesses the child's ability to provide specific, relevant, and detailed relational episodes. “Preoccupied involving anger” takes into account whether and to what extent the child experiences anger toward his/her parents, whereas “Resolution of conflicts” evaluates the child's ability to talk about a conflicting episode with his/her parents while communicating a sense of resolution to the interviewer.

“Idealization” and “Dismissal” refer respectively to the child's tendency to twist the representations of the parents and the relationship with them in a positive direction, and the propensity to minimize the importance of the parents by actively dismissing and/or by derogating them.

According to Shmueli-Goetz et al. (2011), disorganized and/or atypical behavior would emerge during the CAI administration through disorganized, strange, dysregulated, dissociated, or controlling behavior. By taking control or leading the interview, a controlling child could in some instances manifest excessive concern regarding the interviewer and/or the parents who are depicted as weak and helpless.

Positive indices of “Overall coherence” are fresh speech and reflectiveness, whereas negative markers are high rates on “Idealization,” “Dismissal,” “Preoccupied and involving anger,” as well as low rates on “Emotional Openness,” “Balance of positive/negative references to attachment figures,” “Use of examples,” and “Resolution of conflicts.” Generally speaking, incoherent narratives are inhibited, poorly comprehensible, and contain contradictions and inconsistencies.

Coding is based on video-recorded interviews to allow behavioral and linguistic analysis to be carried out. In the CAI it is key to ask the child to recall and to talk about relational episodes (REs) because REs allow us to better understand the mental representations eliciting both linguistic and non-verbal communication. Lastly, an overall classification is assigned distinctly for the mother and the father. According to a two-way classification, the child may be classified as Secure or Insecure, whereas based on a four-way classification he/she may be classified as Secure, Dismissing, Preoccupied, or Disorganized.

Secure children show good emotional openness, balance of positive/negative references, use of examples, a sense of having resolved the conflict, and a coherent narrative. Insecure-Dismissing children rate high on the “Idealization” and/or “Dismissal” subscales, whereas Insecure-Preoccupied children exhibit anxious/depressive preoccupation and/or preoccupied anger. Children who are classified as Dismissing or Preoccupied produce incoherent narrative.

The main attachment classification of Disorganization and a second alternative classification of Secure, Dismissing, or Preoccupied are assigned to subjects in whom disorganized and/or atypical behavior is evident.

Several studies have been conducted since the development of the CAI, but to date and to the best of our knowledge, no reviews containing published empirical research have ever been reported.

The aim of the current paper was to summarize previous empirical investigations in order to (a) provide an overview of the state of the current research, (b) identify unanswered questions, and (c) propose future research directions.

In particular, this review focuses on (1) the psychometric properties of the CAI, (2) alternative interpretative CAI analyses, and (3) the contribution of the CAI to the field of psychology and psychopathology in middle childhood and in adolescence.

## Methods

The decision was made to conduct a narrative review to map the current research findings, thus a structured search strategy was used based on Collins and Fauser (2004) recommendations.

A search for the term “Child Attachment Interview” in titles, abstracts, and in main texts was carried out in the Medline, Scopus, Web of Science, and PsychINFO databases regarding entries from January, 2003 through July, 2016, followed by a search in Mendeley. The search was conducted solely in English language, peer-reviewed journals. However, limits were set to exclude dissertations, chapters in books, and qualitative or theoretical papers. Empirical studies were included if they included the CAI among the measures used in the study, and if they were published in English language, peer-reviewed journals before July, 2016. Unpublished work or data were excluded, as is to be expected in a narrative review.

The search produced 103 studies. After reading all the abstracts, 51 studies were considered to be potentially relevant and were examined, which ultimately led to the inclusion of the 39 studies that actually met the criteria.

## Results

### Psychometric properties of the CAI

#### A brief overview of the CAI validation studies

Very high concordance regarding attachment classification with respect to the mother and father was generally found, ranging—for the four-way classification—from 87.9% in an American clinical sample of adolescents with severe psychopathology (Venta et al., 2014) to 94% in a German community sample of children.

In the first validation study (Target et al., 2003), three sets of the CAI subscales (state of mind, representations of mother and father) were found to be strongly correlated (Cronbach's α = 0.94), but only the “state of mind” set (comprising the subscales “Use of examples,” “Balance,” “Emotional openness,” “Conflict resolution,” and “Coherence”) yielded a high internal consistency (Cronbach's α = 0.92). In the second validation study (Shmueli-Goetz et al., 2008), three further sets of scales were considered depending on the attachment category expected to be linked to each scale, namely “State of mind” (including Coherence, Use of Examples, and Balance), “Active conflict” (comprising Involving Anger and Conflict Resolution), and “Avoidance” (consisting of Emotional Openness, Idealization, and Dismissal) scales. Both “State of mind” and “Avoidance” yielded a high internal consistency (Cronbach's α = 0.87 and 0.82, respectively), whereas the “Active conflict” scale showed low internal consistency (Cronbach's α = 0.32) which was interpreted as being due to the fact that most children did not show Involving Anger with either of their parents, and those who manifested anger toward one parent often had a positive representation of the other parent.

Three later studies investigated the CAI factor structure. Firstly, Zachrisson et al. (2011) performed a confirmatory factor analysis (CFA) on a community sample of Norwegian children aged 9–13 and found that the “Security-Dismissal” one-factor model, as well as a model including the supplementary factor “Preoccupation-Idealization,” fit the data well. Venta et al. (2014) then conducted a maximum likelihood factor analysis using oblique, promax rotation on an American sample of severely disordered adolescents after reversing scores related to anger, dismissing, and idealization subscales to obtain higher scores as markers of security. This led to the extraction of three factors named “Coherence” (including Emotional openness, Balance, Use of Examples, Dismissal, Resolution of Conflict, and Overall Coherence), “Anger” (including Preoccupied Anger with mother and father), and “Idealization” (including Idealization with mother and father). Together, the three factors explained 66.43% of the variance of all 11 scales. Most recently, the study by Borelli et al. (2016c) yielded a two-factor solution in an American community sample of children, which they named DismissingF, with high scores indicating high security/low dismissal (high emotional openness, use of examples, balance of descriptors, and conflict resolution, and low idealization/dismissal of parents) and PreoccupiedF, with high scores signifying high security/low pre-occupation.

#### Inter-rater reliability

Inter-rater reliability was higher in samples of children than in clinical samples of adolescents. In an English mixed (referred and non-referred children) sample it was satisfactory among three expert coders (median ICC for all scales = 0.88; ICCs ranging from 0.38 for Idealization of father to 0.92 for Emotional Openness), as well as between two expert coders (median *r* = 0.87; *r* ranging from 0.66 for Anger with Father to 0.91 for Emotional Openness), and between an undergraduate student given a 3 day training course and an expert coder (median *r* = 0.81; *r* ranging from 0.47 for Anger with father to 0.86 for Coherence and Dismissal of father; Shmueli-Goetz et al., 2008). Inter-rater reliability was also satisfactory in an American community sample of children in which high levels of agreement for four-way classification (*k* = 0.91) and for subscale rating (ICCs ranging from 0.72 to 0.97) were found (Borelli et al., 2016c). In an American clinical sample of adolescents, Venta et al. (2014) found substantial inter-rater reliability with respect to mother (*k* = 0.59; 73.7% agreement for four-way classifications), moderate inter-rater reliability with respect to father (*k* = 0.52; 65.8% agreement for four-way classifications) and an average correlation of 0.66 on all subscales, with *r* ranging from 0.53 (Dismissal of father) to 0.90 (Idealization of father).

Taken together, these findings indicate that coding the scales for the father, especially in a clinical sample, is more challenging. It was assumed that the lower inter-rater reliability that was found for the scales regarding the father might be due to the lack of information about the fathers that was frequently noted in the CAI narratives (Shmueli-Goetz et al., 2008).

### Test-retest reliability

Findings by Shmueli-Goetz et al. (2008) showed high stability coefficients for CAI scales over 3 months (median = 0.69) with noteworthy variability in the temporal stability among the scales (ranging from 0.29 for Involving Anger with father to 0.90 for Involving Anger with mother). The aggregate scales of State of Mind, Active Conflict, and Avoidance revealed satisfactory stability with correlations between 0.69 and 0.78 over 3 months. Test-retest at 1 year showed a decrease in stability (median = 0.54; range 0.08–0.75) with the lowest correlations regarding Idealization and Involving Anger with Father, as well as Idealization of Mother. Notably, the “State of mind” scale maintained good stability both across a 3 month (*r* = 0.78) and 1 year (*r* = 0.71) interval, as did the overall classification (Cohen's *k* ranging from 0.52 to 1.00 at 3 months, and from 0.42 to 0.74 at 1 year). The percentage of agreement after 1 year was 76% for the mother and 69% with respect to the father for the four-way classification.

#### Discriminant validity

No significant effect related to verbal IQ and expressive language, or any effects regarding socioeconomic status (SES), ethnicity, and one-or two-parent household were found in either community or referred samples (Shmueli-Goetz et al., 2008). Furthermore, a very recent study (Borelli et al., 2016c) found no significant associations among CAI factors and scores obtained on three temperamental scales regarding shyness, fear, and inhibitory control as reported by parents. Although most studies did not find a significant association between gender, age, and attachment security in middle childhood, a significant effect of gender was observed in a referred sample of children (Shmueli-Goetz et al., 2008) as well as in two community samples of children (Zachrisson et al., 2011; Borelli et al., 2016c), with boys more frequently being classified as Insecure-Dismissing than girls.

Evidence of divergent validity was also provided by a recent study (Borelli et al., 2016d) which investigated whether the CAI narrative is a unique measure of the state of mind regarding attachment, rather than a measure of the general speech style. Findings supported the specificity of the children's discursive style and linguistic behavior during the CAI when compared to speech used during a Non-Relational Interview (NRI). In addition, only CAI coherence (not NRI coherence) predicted cardiovascular reactivity to a relational probe.

#### Convergent validity

Some studies investigated the CAI convergent validity using semi-projective as well as self-report measures of attachment. Shmueli-Goetz et al. (2008) found a 64% agreement rate (*k* = 0.36; *p* < 0.005) for three-way classifications between the CAI and Separation Anxiety Test (SAT; Wright et al., 1995).

The Kerns Security Scale (KSS; Kerns et al., 1996), The Inventory of Parent and Peer Attachment (IPPA; Armsden and Greenberg, 1989), and the Parental Bonding Instrument (PBI; Parker et al., 1979) were used in two studies to explore the convergent validity of CAI classifications and subscales and self-report measures of attachment. Although one of the studies was conducted on a community sample of children (Borelli et al., 2016c) and the other was carried out on a clinical sample of adolescents (Venta et al., 2014), they both yielded similar findings. CAI coherence correlated significantly positively with self-reported security and negatively with self-reported overprotection, especially with respect to the mother. Children who were classified as secure reported the highest security, while children who were classified as Pre-occupied reported the lowest indexes of security. In the clinical sample, Idealizing children obtained high scores on the self-reported measure of attachment security, and they reported high levels of warmth and care in their relationships with their parents, especially with the mother (Venta et al., 2014). The CAI disorganized classification did not correlate with the self-report measure of attachment, thus raising questions about how disorganization of attachment manifests itself during adolescence. It was argued that during adolescence, Disorganized children develop a controlling stance on parents which enables them to manage caregiver behavior, making it possible for the adolescent to regulate his/her emotions and actions (Venta et al., 2014).

Taken together, the findings from validation studies showed moderate correlations between the CAI and projective and self-report measures of attachment, thereby supporting the notion that the CAI taps into a distinctive, albeit partially overlapping, construct.

Noteworthy, the association between the CAI and self-reported attachment measures was only statistically significant along the secure and the preoccupied dimensions, whereas it was found neither with regard to dismissing nor to disorganized attachment. Findings suggested that the CAI is a unique measure for distinguishing secure from dismissing children as well as for identifying disorganized children, even though the CAI coding system could be improved as far as the detection of disorganization is concerned. In the clinical setting, making these distinctions could be essential for deciding intervention, and it is also crucial for an in-depth study of the implications of the various organized attachment strategies and the developmental pathways of disorganization.

It is remarkable that the convergent validity was assessed almost exclusively using questionnaire measures which are not comparable on a theoretical level to the CAI that is a representational attachment measure. Nevertheless, the issue of variability in methodologies that are used for assessing attachment in different developmental ages (Madigan et al., 2016) made it difficult to use other representational attachment measures in CAI convergent validity studies. Currently, indeed the few available representational attachment measures are designed to assess attachment in defined age groups, so it would be possible to study CAI convergent validity with other representational measures such as the Manchester-Child Attachment Story Task (MCAST; Green et al., 2000) and the Story Stem Assessment Profile (SSAP; Hodges et al., 2003) only in samples of children aged 7–9, because these latter measures were designed to be used with young children alone.

#### Predictive validity

Secure children showed better social adaptation (Shmueli-Goetz et al., 2008), they reported fewer externalizing symptoms, both self- and parent-reported (Venta et al., 2014), as well as fewer depressive symptoms (Venta et al., 2014; Borelli et al., 2016c) and higher emotion control (Borelli et al., 2010a, 2016b). In particular, preoccupied children reported more depressive symptoms (Borelli et al., 2016c), while idealizing children reported less externalizing psychopathology (Venta et al., 2014), as well as fewer depressive symptoms (Borelli et al., 2016c), thus confirming their marked tendency to minimize experiences of distress. These findings supported the notion that self-report measures are not efficient in distinguishing an idealizing state of mind regarding attachment due to the marked tendency of Idealizing individuals to normalize and embellish their experience. Anxiety disorders did not correlate with attachment security in a community sample of children (Borelli et al., 2016c) or in a sample of adolescent inpatients (Venta et al., 2014), thus leading to the hypothesis that the pathway to anxiety disorders is a complex one that does not involve attachment security alone (Venta et al., 2014). Secure children had high scores on Extraversion and lower scores on an aggression self-reported measure, while dismissing children reported the highest level of both direct and indirect aggression, and preoccupied children scored high on Neuroticism (Roder et al., 2015). Unexpectedly, Venta et al. (2014) found a limited association between attachment security, thus hypothesizing that it could be due to the developmental phase of transition and/or to the possibility that peer relations are regulated by a behavioral system different from attachment. A significant association (*k* = 0.16; *p* < 0.002) was found between the child's and the mother's attachment pattern (assessed by the AAI) on the four-way classifications, offering further support to the predictive validity of the CAI.

#### Incremental validity

To our knowledge, the CAI incremental validity has, to date, only been investigated in one study (Borelli et al., 2016c), which explored the contribution of the CAI in predicting children's anxiety and depressive self-reported symptoms above and beyond self-report measures. Findings support the CAI incremental validity in predicting children's depressive symptoms. Higher preoccupied attachment predicted greater self-reported depressive symptoms, while higher dismissing attachment predicted fewer self-reported depressive symptoms. Both CAI factors and Kerns Security Scales uniquely contributed to predicting the children's depressive symptoms, albeit KSS explained a greater portion of variance, but not anxiety symptoms. Only a marginal association was found between the CAI pre-occupied factor and anxiety, while no association was found between the latter and the KSS scores. Taken together, findings from this study indicate that both the CAI and the KSS may provide complementary information about internalizing disorders in middle childhood.

### Alternative interpretative CAI analyses

Unlike the usual top-down approach, some studies used a bottom-up, word-count-based analysis of the CAI narrative to evaluate linguistic correlates of the attachment state of mind as well as to investigate mental state talk in the context of autobiographical narratives.

Borelli et al. (2011) applied a computerized text analysis to the CAI transcripts to verify the hypothesis according to which specific patterns of discourse are associated with the four different classifications of the state of mind regarding attachment. In particular, they investigated verbal immediacy (i.e., the use of concrete, personal, involved, experiential language with a focus on the here and now), thought to be a marker of the extent to which an individual feels experientially connected to what he is talking about. Their hypothesis, according to which children's attachment interviews differ in verbal immediacy as a function of their attachment classification, received strong support. Specifically, dismissing individuals reported the lowest level of verbal immediacy, whereas Pre-occupied children showed the highest level, and disorganized children showed decreasing verbal immediacy during discussion of loss, and a higher occurrence of words related to loss in non-loss sections of the CAI. More recently, Borelli et al. (2016d) found a significant association between the mother-child relational language style matching and child security.

Scopesi et al. (2015) analyzed the CAI transcripts using a word-count-based analysis to investigate the relationship between children's and maternal mental state talk in the Adult Attachment Interview (AAI; George et al., 1985) and found a significant correlation between the maternal and the child's use of markers of uncertainty in the context of attachment narratives. A following study (Rosso et al., 2015) offered further support to the notion that maternal reflective functioning, rather than maternal attachment security, predicted the child's mental state talk in the CAI transcripts.

CAI transcripts were also analyzed in two further studies by using the qualitative technique of the Interpretative Phenomenological Analysis (Smith, 2004; Smith et al., 2009). One study investigated the main themes concerning young women's experiences regarding their obesity (Holland et al., 2012), while the second study focused on the parenting styles that were adopted by birth and foster parents in a sample of abused or neglected children placed in foster care (Ahmed et al., 2015).

As regards the AAI, the DMM (Dynamic Maturational Model of Attachment; DMM, Crittenden and Landini, 2011) was also applied to CAI coding (Farnfield, 2014). This study, which was the only one of its kind, involved a relatively small sample of children (*n* = 41). It yielded promising results in identifying the full range of DMM attachment strategies and showed a high level of agreement between coders. Findings showed that the DMM might be useful in further investigating how disorganization of attachment develops from infancy to middle childhood and adolescence, especially in high risk samples.

### The contribution of the CAI to psychology and psychopathology in middle childhood and in adolescence

#### The distribution of attachment patterns in middle childhood and in adolescence

Table 1 provides a summary of the reviewed studies, reporting the type of sample, the children's age, and the attachment pattern distribution. Although the CAI was initially developed to investigate attachment patterns in children aged 7–12 years, the most recent studies, including a validation study (Venta et al., 2014), comprised adolescents up to 17 years of age. Regarding the distribution of the attachment patterns, in the most recent studies these data were not available because of the preferred use of the dimensional vs. the categorical approach. When available, the distribution revealed a prevalence of the dismissing pattern among the insecure patterns, whereas the preoccupied pattern was notably underrepresented both in clinical and in community samples.

**Table 1:** **Sample size, sample type, age, and attachment distribution in the reviewed studies**.

	***n***	**Age**			**Sample type**	**Attachment pattern**			
		**range**	***M (SD)***			**Secure**	**Dismissing**	**Preoccupied**	**Disorganized**
Ahmed et al., 2015	12	13–15	N.A.		Fostered	Not available, other interpretative system used			
Bizzi et al., 2015	20	8–15	11.35 (1.90)		Disruptive behavior disorders	20%	25%	10%	45%
	20	8–15	11.99 (2.25)		Somatic symptoms disorders	20%	30%	5%	45%
Borelli et al., 2010a	97	8–12	10.01 (1.52)		Community	44%	31%	6%	19%
Borelli et al., 2010b	Overlapping (Borelli et al., 2010a)								
Borelli et al., 2013	Overlapping (Borelli et al., 2010a)								
Borelli et al., 2016a	117	8–12	9.8 (1.46)		Community			Not available	
Borelli et al., 2011	Overlapping (Borelli et al., 2010a)								
Borelli et al., 2016c	104	8–12	9.8 (1.46)		Community			Not available	
Borelli et al., 2016d	125	8–12	9.8 (1.46)		Community	64.8%	17.6%	9.6%	8%
Borelli et al., 2014	106	8–12	9.83 (1.49)		Community	63%	20%	9%	8%
Ensink et al., 2016	74	7–12	N.A.		Sexually abused			Not available	
	94	7–12	N.A.		Community			Not available	
Ensink et al., 2015	22	N.A.	9.3 (1.5)		Intrafamilial abuse			Not available	
	24	N.A.	10 (1.5)		Extrafamilial abuse			Not available	
	48	N.A.	9.11 (1.4)		Community			Not available	
Farnfield, 2014	24	7–11	N.A.		Community			Not available	
	17	7–11	N.A.		Fostered			Not available	
Fearon, et al., 2014	1102		13.9–16.4		Community	52.7%	39%	5.4%	2.9%
Fox and Borelli, 2015	30	8–12	N.A.		Children of depressed mothers			Not available	
Glazebrook et al., 2015	51	13–17	N.A.		Self-harm	27.5%		Not available	
Holland et al., 2012	8	13–16	N.A.		Obese females			Not available	
Humfress et al., 2002	71	12–13	12.7 (N. A)		Community			Not available	
Jardin et al., 2015	229	12–18	N.A.		Inpatient	26.2%		Not available	
Joseph et al., 2014	62	10–17.	13.86 (1.95)		Fostered	9%	55%	2%	35%
	50	10–17	14.19 (1.65)		Community	60%	18%	22%	–
Roder et al., 2015	72	N.A.	10 (0.48)		Community	70.9%	22.3%	3.9%	5.9%
Rosso et al., 2015	41	12	N.A.		Community			Not available	
Scopesi et al., 2015	Overlapping (Rosso et al., 2015)								
Scott et al., 2011	102	9–17		N.A.	High risk	52%		Not available	
	96	9–13		N.A.	Moderate risk	73%		Not available	
	50	10–17		N.A.	Normative risk	60%		Not available	
Sharp et al., 2016	259	12–17		N.A.	Inpatient			Not available	
Shmueli-Goetz et al., 2008	161	7–12		10.9 (1.9)	Community	66%	28%	3%	4%
	65	7–12		10.4 (1.2)	Clinical	30%	50%	11%	9%
Stern et al., 2015	58	7–12		9.9 (1.5)	Community	76%	17%	7%	N.A.
Target et al., 2003	161	7–12		11.1 (1.6)	Community			Not available	
	65	7–12		10.2 (1.3)	Clinical			Not available	
Tessier et al., 2016	39			9.5 (1.6)	Abused			Not available	
	21			9.0 (1.2)	Community			Not available	
Venta and Sharp, 2014	Overlapping (Venta et al., 2014)								
Venta et al., 2015a	194	N.A:		15.4 (1.4)	Inpatient	25.3%		Not available	
Venta et al., 2015b	240	12–17		N.A.	Inpatient	24.2%		Not available	
Venta et al., 2014	194	12–17		N.A.	Inpatient	30.4%	38.1%	14.4%	17%
Vorria et al., 2015	52	12–13		13.1 (.05)	Adopted	50%	N.A.	N.A.	6%
	36	12–13		13 (.05)	Community	64%	N.A:	N.A:	0
White et al., 2013	Overlapping (Borelli et al., 2010a)								
White et al., 2012	Overlapping (Borelli et al., 2010a)								
Zaccagnino et al., 2015	24	10–13		11.2 (1.7)	Fostered	8.7%	82.6%	0	8.7%
	35	9–13		10.7 (0.8)	Community	62.9%	22.8%	8.6%	5.7%
Zachrisson et al., 2011	150	9–13		11.7 (1.3)	Community	63.3%	31.3%	3.3%	0.7%

Under-representation of the preoccupied pattern in middle childhood did not allow for a more in-depth investigation of this attachment strategy, whereas the relatively frequent occurrence of the dismissing pattern provided the opportunity to explore whether the dismissal attachment strategy involves a specific way to regulate emotions by minimizing their importance and awareness, even in a non-familial context, as was largely observed in adults. Studies found that dismissing children manifested a marked tendency to underreport their subjective distress, showing a greater discrepancy between subjective and physiological emotional response (White et al., 2012, 2013; Borelli et al., 2014), even in a non-relational context (Borelli et al., 2013). Despite their tendency toward a positive bias in perceiving their parents as more loving (Borelli et al., 2013), they showed a higher expectation of rejection by peers (White et al., 2012, 2013). These findings should be kept in mind when interpreting data from child-report measures of parental care and emotional experience. Furthermore, as Borelli et al. (2013) suggested, future studies are needed to explore the development of the different dismissing strategies, namely those based on idealization and those based on the dismissal of the attachment figures as well as the dismissal of one's own attachment needs.

#### Attachment security and mentalization in middle childhood and in adolescence

The crucial role of parental reflective functioning in the intergenerational transmission of attachment security during early childhood has been firmly supported by an extensive body of research (e.g., Grienenberger et al., 2005; Slade et al., 2005). On the other hand, the research in this field has just begun with regard to middle childhood and adolescence. To the best of our knowledge, only one recently published study (Borelli et al., 2016a) investigated the link between parental reflective functioning (measured by the Reflective Functioning Scale applied to the Parent Development Interview, PDI-RF, Slade et al., 2004) and attachment security during middle childhood. Findings showed that primary caregivers of dismissing children reported the lowest RF, and that only child-focused RF (not parental self-focused RF) was significantly associated with the child's attachment security, as dimensionally assessed by the CAI Coherence scale. An earlier study (Stern et al., 2015) focused on the role of parental empathy in the transmission of attachment security. According to Stern et al., empathy differs from RF in that it refers to a parent's in-the-moment experiences of emotional attunement with the child during which the parent's emotional experience reverberates the child's emotions, focusing specifically on the child's experiences of distress or need. In this perspective, RF was considered a metacognitive ability that is essential but not enough for empathic parenting. Findings showed a significant association between parental empathy, child attachment security (assessed by the continuous score for narrative coherence), emotional openness on the CAI, and child perceptions of parental warmth.

Furthermore, the development of the CAI provides researchers with an instrument to investigate the link between attachment security and mentalization ability beyond early childhood. An early study (Humfress et al., 2002) found a significant association between attachment security and mentalization in a community sample of early adolescents. A version of the Reflective Functioning Scale, which was originally developed for the AAI (ARFS; Fonagy et al., 1998), was then revised to be applied to the videotaped and transcribed CAI narratives (CRFS: Ensink, 2004; Ensink et al., 2013), to search for markers of the child's mentalization in the descriptions of himself/herself and of his/her relationships with their attachment figures. As for the use in the AAI, four markers indicate reflective functioning in the CAI transcripts, namely “Awareness of the nature of mental states” (marker A), “Explicit effort to tease out mental states underlying behavior” (marker B), “Recognizing developmental aspects of mental states” (Marker C), and “Mental states in relation to the interviewer” (Marker D). The first studies using the CRFS were conducted on samples of abused children. Sexually abused children showed lower reflective functioning than non-abused children (Ensink et al., 2015; Tessier et al., 2016), especially when they experienced intrafamilial abuse (Ensink et al., 2015). In addition, Ensink et al. (2016) found that children who experienced sexual abuse suffered from depressive and externalizing symptoms to a lesser extent if they were able to mentalize in the context of the CAI narrative.

Furthermore, the association between attachment security and mentalization deficit was investigated using an independent measure of mentalization in a sample of 259 adolescents admitted to an inpatient unit, and findings showed that hypermentalizing was significantly correlated with attachment insecurity, assessed dimensionally as CAI narrative coherence (Sharp et al., 2016).

#### Attachment in maltreated children

Some studies investigated the state of mind regarding attachment in adolescents who suffered traumatic experiences such as severe neglect, maltreatment, and sexual abuse during childhood. Taken together, these studies found that although these children mostly manifested insecure and disorganized attachment representations during the period of residential care and the early stage of foster care placement (Vorria et al., 2015; Zaccagnino et al., 2015), they frequently developed a subsequent secure attachment pattern toward their foster parents (Joseph et al., 2014; Vorria et al., 2015). They were able to have distinct representations of the different parenting received from their birth and foster parents (Ahmed et al., 2015), although they maintained insecure state of mind regarding attachment toward their biological parents (Joseph et al., 2014). A further study (Jardin et al., 2015) found that attachment security shielded adolescents who experienced sexual trauma against the later occurrence of trauma symptoms.

#### Attachment and psychopathology in middle childhood and in adolescence

A recent meta-analysis (Madigan et al., 2016) strongly supported the notion that attachment insecurity in children aged 3–18 years was associated with internalizing and externalizing symptoms. Specifically, it was found that internalizing behavior was associated with all insecure attachment patterns, while only disorganized attachment was associated with externalizing behavior.

The development of the CAI allowed researchers to explore the association of attachment insecurity and psychopathology distinctively in middle childhood as well as in adolescence. Some studies (Scott et al., 2011) confirmed that attachment security in adolescence explained unique variance in adjustment, regardless of other components of the parent-adolescent relationship. Security attachment was also found to be a protective factor against the onset of depression in the children of depressed mothers because of the ability of secure children to regulate emotions as well as to adopt successful coping strategies throughout periods of stress (Fox and Borelli, 2015). In general, a prevalence of insecurely attached children was found in clinical samples (Target et al., 2003; Shmueli-Goetz et al., 2008; Venta et al., 2014; Bizzi et al., 2015; Glazebrook et al., 2015; Jardin et al., 2015). Disorganized attachment was associated with child experiences of depressive symptoms and shyness, and with parent-reports of social anxiety, inattention, and thought problems (Borelli et al., 2010b) as well as with disruptive behavior and Somatic Symptoms Disorders (SSDs; Bizzi et al., 2015). A dismissing pattern was the most common finding in a very small sample of eight obese adolescent females who had taken on a caring role in their families, dismissing their own vulnerability and emotional needs and thus preserving self-reliance (Holland et al., 2012).

Discordant findings were found in samples of adolescents with self-harm and suicide-related thoughts and behaviors. Venta and Sharp (2014) did not find any significant relationship between attachment organization and suicide-related thoughts and behaviors, whereas Glazebrook et al. (2015) found that insecure adolescents were more likely to repeat self-harm 6 months later; conversely, secure adolescents significantly improved their problem-solving skills. Venta and Sharp argued that their discrepant results might be due, at least partially, to the general severity of psychopathology in their sample which was characterized by a very high base-rate of suicide-related thoughts and behaviors.

With regard to the outcomes, Venta et al. (2015a) found a greater decrease in internalizing symptoms in securely attached adolescents, whereas the organization of attachment did not explain variations in externalizing behaviors. Furthermore, they found that the association between attachment security and reduction in internalizing symptoms was mediated by emotion regulation, namely the propensity of secure adolescents to react negatively to negative emotions to a lesser extent than insecurely attached adolescents.

#### The role of genes in attachment in adolescence

A recent study (Fearon et al., 2014) found a significant genetic influence—as well as a substantial influence of the non-shared environment—on adolescent attachment, in contrast to findings from twin studies conducted during early childhood that found a very limited genetic influence on attachment security (e.g., Bokhorst et al., 2003). Fearon et al. found a strong association between twins' attachment security only in monozygous (MZ) adolescent twins, while significantly lower associations emerged in the dizygous (DZ) twins group. These findings prompted some questions regarding the possibility that (a) genes might have an impact on later attachment organization, exerting progressively more influence on parenting style which, in turn, contributes to the quality of the parent-child relationship, and (b) the genes exert a greater influence on the representational ability which is required to evaluate security of attachment in adolescence. Actually, the CAI bases its classification of attachment security on a representational level, rating the adolescent's ability to think coherently about, and reflect upon, his/her attachment experiences, whereas attachment security in early childhood is measured at a behavioral level. Furthermore, this study highlighted the complexity of the intergenerational transmission of attachment security beyond early childhood, also suggesting that the non-shared environmental mechanisms implicated in attachment organization should be a central topic in further studies.

## Discussion

A review of the empirical studies involving the CAI that have been published in English language, peer-reviewed journals pointed out and raised a number of relevant open issues. The most critical aspects concerned the CAI coding and classification system. As discussed below, some relevant questions arose regarding (a) how opportune it would be to maintain a distinct classification for mother and father, (b) coding challenges regarding the father as well as the Pre-occupied and Disorganized classification, and finally c) the advantage of a dimensional vs. a categorical approach.

### Concordance between attachment to mother and father

Substantial concordance between attachment to mother and father was found in all the reviewed studies. Shmueli-Goetz et al. (2008) argued that individuals in middle childhood seemed to show an increased integration of internal working models with respect to mother and father, thus they suggested using a single index of security based on the continuous measure derived from the rating scales. Noteworthy, a recent study (Di Folco et al., 2017) found concordance of representations of attachment (assessed by MCAST) to mother and to father even in children aged 6–7 years. Venta et al. (2014) also suggested using the coherence subscale as a measure of overall attachment security after finding that it correlated significantly with attachment self-report measures for both parents. Nevertheless, two separate classifications as well as distinct subscales with respect to mother and father allow a more in-depth investigation of attachment in middle childhood and in adolescence. Since longitudinal studies are currently lacking, it might be interesting to investigate whether internal working models with respect to each parent change from middle childhood to adulthood, as well as whether and how they evolve based on gender.

### Coding challenges regarding the father

Validation studies yielded lower inter-rater reliability for the father as well as lower temporal stability, especially regarding the attachment classification and the subscale rates with respect to the father. It was argued that children might find it more difficult to talk about their relationship with their father, showing a frequent tendency to limit their recounts to facts and activities. It was also believed that they might be more strongly influenced by their more recent experiences than adults are when depicting their close relationships and that they could be highly influenced by the actual changes in the family (Shmueli-Goetz et al., 2008). In addition, Venta et al. (2014) argued that attachment may be less stable in adolescence than in adulthood as the former is a developmental phase of transition. It could be questioned whether the mental representation of the relationship with the father might be more subject to variations during adolescence. Therefore, future studies addressing these issues are needed, with a focus on gender differences as well.

### Relatively high frequency of secure children in clinical samples and under-representation of preoccupied and disorganized children in community as well as in clinical samples

According to Shmueli-Goetz et al. (2008) the 25–30% rate of individuals classified as Secure in the clinical samples could raise some questions regarding both the insensitivity of the measure and heterogeneity in the clinical samples. They noted that most of the secure children in the clinical samples coherently described negative and disturbed relationships with their parents. Consequently, the authors questioned whether secure children in the clinical samples achieved a somewhat forced coherence in their attachment representations, which in turn contributes to an impairment in the psychic functioning on another level. Therefore, additional coding of the Current Experiences may be needed in the CAI coding system together with more in-depth studies of secure narratives which describe negative close family relationships. Venta et al. (2014) did not believe that the presence of a considerable percentage of secure children in their inpatient sample was a consequence of an inadequacy of the measure since about one fourth of the adults in the clinical samples also showed attachment security. On the contrary, they argued that attachment insecurity may be only one pathway among several to psychopathology and that it is not a necessary precondition to psychopathology, although it is quite a common occurrence. Regarding this issue, a fascinating study (Venta et al., 2015b) investigated the construct of earned attachment security (Pearson et al., 1994) in a sample of 240 inpatient adolescents. They found that 19 (8%) adolescents who were assigned to the secure CAI classification reported negative experiences with their mothers on the care subscale of the Parental Bonding Inventory (PBI; Parker et al., 1979). The finding that this subsample of secure adolescents showed a significantly higher lack of emotional awareness than the continuous secure group led to some interesting hypotheses regarding the role of effortful control as well as of mentalization in determining a secure attachment narrative, even in the presence of recall of negative attachment experiences. It could also be questioned whether and to what extent low emotional awareness can be a psychological resource or rather a defensive liability preventing these children from feeling and expressing anger as well as being over-involved in painful experiences, thus receiving a secure classification instead of a preoccupied one. Actually, a further finding of the present review was that only a few children were rated as preoccupied. Could this finding be due to the difficulty of the measure to detect preoccupation? Children may manifest a preoccupied state of mind differently from adults; involving anger was a rare finding, and pre-occupied children communicate negativity, repetitive themes, and depressive mood even in non-verbal behavior. Shmueli-Goetz et al. (2008) wondered whether these children might have been erroneously classified as Secure because of their ability to provide examples, their emotional openness, and fairly coherent narratives. To overcome this limitation, there was a proposal to add a further scale to the CAI coding system which depicts the anxious preoccupied communicative patterns, also extending and further detailing relevant non-verbal behavior (Shmueli-Goetz et al., 2008; Venta et al., 2014). The coding and classification manual was recently revised, thus the current version (Version VI-November 2011) includes additional indicators of an anxious depressive ruminative preoccupation, although it is not an additional scale (Shmueli-Goetz et al., 2011).

This recent version also significantly extended the description of the disorganized pattern, since previous studies highlighted some possible inadequacies in this regard. Notably, Disorganized children had mothers who showed an unresolved state of mind regarding loss or trauma in their AAI narratives, but most mothers who were classified as Unresolved by the AAI had children who were not coded as Disorganized. These findings, together with the evidence of a low frequency of the Disorganized category (<10% of cases) in the referred sample, raised doubts about the sensitivity of the CAI in detecting disorganization (Shmueli-Goetz et al., 2008). Furthermore, the CAI disorganized classification did not correlate with self-report measures of attachment, thus raising questions about the ways in which disorganization of attachment manifests itself during adolescence. It was argued that during adolescence, disorganized children develop a controlling stance on parents which enables them to manage caregiver behavior, making it possible for the adolescent to regulate his/her emotions and actions (Venta et al., 2014). Future studies are needed to further explore these issues by taking advantage of the recently revised coding guidelines.

### Dimensional vs. categorical approach

A dimensional approach was preferred to a categorical one since factor scores provide higher statistical power to detect differences while still preserving the distinction between different insecure attachment strategies. Some studies investigated the CAI factor structure, and three factor structures have been identified to date (Zachrisson et al., 2011; Venta et al., 2014; Borelli et al., 2016c).

Venta et al. (2014) believed that further taxometric analyses would be necessary to investigate whether a dimensional or a categorical model best explains differences in attachment security in adolescence. Until future studies actually do shed light on this topic, they suggest using both a categorical and a dimensional approach using the three-factor analytically-derived subscales they identified in their study, since they explained most of the variance in all the CAI subscales while lessening the number of variables. Borelli et al. (2016c) stated the need for a future scale to evaluate the disorganized pattern instead of the sole, currently existing categorical measure of disorganized attachment, so that a more comprehensive factor structure could be tested.

## Conclusions and future directions

The present review shows that the CAI prompted extensive research into the field of developmental psychology in middle childhood, thus providing a worthwhile contribution to the investigation of the influence of the parent-child relationship in developmental phases beyond infancy and early childhood. It proved to be a reliable instrument to assess attachment organization in middle childhood and adolescence. Nonetheless, some issues require further investigation. First, CAI coding and factor structure need to be explored more at length. Additional and more in-depth investigation, especially in clinical samples, is needed to understand the peculiar ways that preoccupied involving anger and attachment disorganization are expressed during middle childhood and adolescence. In this view, testing the recently revised, manualized guidelines regarding the indicators of anxious-depressive ruminative preoccupation and attachment disorganization is warranted before investigating the CAI factor structure again. Then, longitudinal studies should explore the development trajectories from childhood to adulthood of preoccupied as well as of disorganized children, which is a clinically relevant issue. Secondly, further studies should address the issue concerning the unsatisfactory inter-rater reliability regarding the classification with respect to the father in order to explore the causes, and then to improve the coding guidelines. Thirdly, longitudinal studies are needed to also investigate the change in mental representation of the relationship with the father from childhood to adolescence and early adulthood, maintaining a focus on gender differences. Finally, studies in clinical settings are still lacking, although the CAI seems to be a potential, clinically relevant measure of attachment in middle childhood and adolescence that could be used to set up prevention, treatment, and outcome studies. It could also prove to be a useful instrument in forensic child custody evaluations, a setting which requires the use of reliable and valid psychological assessment measures.

## Author contributions

The author confirms being the sole contributor of this work and approved it for publication.

### Conflict of interest statement

The author declares that the research was conducted in the absence of any commercial or financial relationships that could be construed as a potential conflict of interest.
